# Maladie de Best

**DOI:** 10.11604/pamj.2019.34.61.19771

**Published:** 2019-10-01

**Authors:** Yasmine Chaoui Roqai, Khanaouchi Nawal

**Affiliations:** 1 Service d’Ophtalmologie, Hôpital Militaire d’Instruction Mohammed V, Rabat, Maroc

**Keywords:** Maladie de best, dystrophie maculaire, enfant, Best's disease, macular dystrophy, child

## Abstract

Best's disease is an inherited macular dystrophy with autosomal dominant inheritance pattern, characterized by the presence of autofluorescent vitelliform deposition whose development is stereotyped from its manifestation to the fragmentation of the material and then to its resorption. Age at onset is between 7 and 12 years. Except in the presence of complications, patients are more often asymptomatic and the disease is discovered fortuitously. We report the case of 8 year-old only daughter with no particular past medical history. Ophthalmologic examination showed corrected visual acuity 7/10 in the left and right eyes, the examination of the anterior segment was normal, fundus examination showed macular yellowish-white stain (A, B). On fluorescein angiography, hyperfluorescence occurred early followed by a hypofluorescence. Macular imaging by optical coherence tomography (OCT) showed the presence of an empty optical space between the neuroretina and the pigment epithelium (C, D). Assessment was performed: electrooculogram was perturbed using Arden ratio of 146% in the right eye and 179% in the left eye. Electroretinogram, visual fields and color vision test were normal. Fundus examination of parents was normal. The diagnosis of previtelliform BEST's disease was made based on early hypofluorescence, OCT appearance and alteration of the electroretinogram. Differential diagnoses included: stargardt's disease, progressive dystrophy of the cones and X-linked retinoschisis. No treatment was proposed, only simple monitoring.

## Image en médecine

La maladie de Best est une dystrophie maculaire héréditaire transmise sur le mode autosomique dominant, caractérisée par la présence de dépôts vitellins auto-fluorescents dont la séquence évolutive est stéréotypée de l'apparition à la fragmentation du matériel jusqu'à sa résorption. L'âge d'apparition compris entre 7 et 12 ans. Le plus souvent de découverte fortuite car asymptomatique sauf en présence de complications. Nous rapportons le cas d'une patiente âgée de 8 ans, fille unique, sans antécédents pathologiques particuliers. L'examen ophtalmologique retrouve une acuité visuelle corrigée à 7/10 ODG, l'examen du segment antérieur est sans particularités, l'examen du fond d’œil retrouve un foyer maculaire blanc-jaunâtre (A, B). L'angiographie à la fluorescéine a objectivé une hyperfluorescence aux temps précoce suivie d'une hypofluorescence. La tomographie par cohérence optique (OCT) maculaire montre la présence d'un espace optiquement vide entre la neuro-rétine et l'épithélium pigmentaire (C, D). Un bilan a été réalisé: l'électro-oculogramme était perturbé avec un coefficient d'Arden à 146% au niveau de l’œil droit et à 179% au niveau de l’œil gauche. L'électro-rétinogramme, les champs visuels, et le test de vision des couleurs étaient normaux. Le fond d’œil des parents était normal. Le diagnostic de maladie de BEST a été posé chez cette patiente au stade pré-vitelliforme devant l'hypofluorescence aux temps précoce, l'aspect de l'OCT et l'altération de l'élécto-rétinogramme. Les diagnostics différentiels pouvant être évoqués sont la maladie de Stargardt, la dystrophie progressive des cônes et le rétinoschisis lié à l'X. Aucun traitement n'a été proposé pour cette patiente mais une simple surveillance.

**Figure 1:**
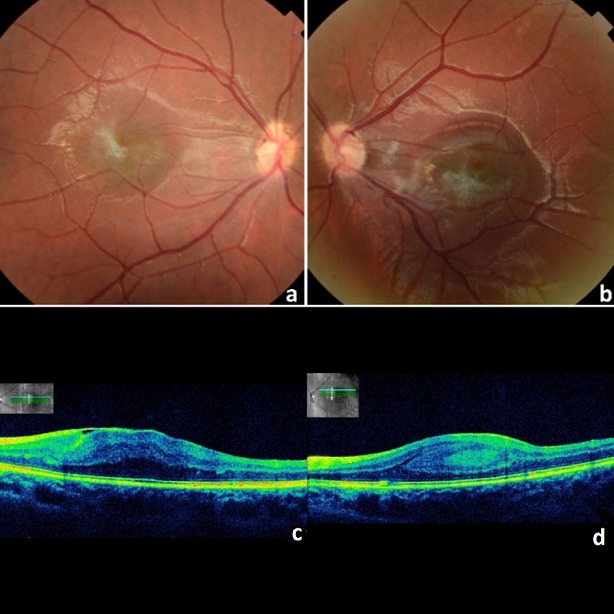
A) rétinophoto de l´œil droit; B) rétinophoto de l´œil gauche; (C, D) oct de l´œil gauche

